# Classification of rare land cover types: Distinguishing annual and perennial crops in an agricultural catchment in South Korea

**DOI:** 10.1371/journal.pone.0190476

**Published:** 2018-01-25

**Authors:** Christina Bogner, Bumsuk Seo, Dorian Rohner, Björn Reineking

**Affiliations:** 1 Ecological Modelling, BayCEER, University of Bayreuth, Bayreuth, Germany; 2 Biogeographical Modelling, BayCEER, University of Bayreuth, Bayreuth, Germany; 3 Institute of Environmental Research, Kangwon National University, Chuncheon, Republic of Korea; 4 Land Use Change and Climate Research Group, Institute of Meteorology and Climate Research – Atmospheric Environmental Research, Karlsruhe Institute of Technology, Garmisch-Partenkirchen, Germany; 5 Chair of Applied Computer Science III (Robotics and Embedded Systems), University of Bayreuth, Bayreuth, Germany; 6 UR LESSEM, Irstea, Université Grenoble Alpes, St-Martin-d’Hères, France; University of Maryland at College Park, UNITED STATES

## Abstract

Many environmental data are inherently imbalanced, with some majority land use and land cover types dominating over rare ones. In cultivated ecosystems minority classes are often the target as they might indicate a beginning land use change. Most standard classifiers perform best on a balanced distribution of classes, and fail to detect minority classes. We used the synthetic minority oversampling technique (smote) with Random Forest to classify land cover classes in a small agricultural catchment in South Korea using modis time series. This area faces a major soil erosion problem and policy measures encourage farmers to replace annual by perennial crops to mitigate this issue. Our major goal was therefore to improve the classification performance on annual and perennial crops. We compared four different classification scenarios on original imbalanced and synthetically oversampled balanced data to quantify the effect of smote on classification performance. smote substantially increased the true positive rate of all oversampled minority classes. However, the performance on minor classes remained lower than on the majority class. We attribute this result to a class overlap already present in the original data set that is not resolved by smote. Our results show that resampling algorithms could help to derive more accurate land use and land cover maps from freely available data. These maps can be used to provide information on the distribution of land use classes in heterogeneous agricultural areas and could potentially benefit decision making.

## Introduction

Detailed information on land use and land cover (lulc) is essential in many areas of environmental sciences. A constantly growing body of literature emphasizes the impact that changes in land use can have on Earth’s climate, biodiversity and water cycle [1–3]. Among different human land use forms, croplands and pastures are particularly frequent and occupy 40% of the land surface [1].

Detecting lulc changes in agricultural areas might be challenging because global land cover databases like GlobCover or modis Land Cover Type Product (MCD12Q1) have few crop-related classes. To derive more detailed lulc maps, time series from the Moderate Resolution Imaging Spectroradiometer (modis) can be used because they allow to track seasonal variation of vegetation development. Several studies have shown that time series could better discriminate between different types of vegetation than single snapshots [4, 5].

Agricultural areas are often fragmented and have a mosaic-like structure. They frequently comprise major and minor land cover types. The latter are often difficult to classify. Indeed, it is commonly recognized that unevenly distributed classes could deteriorate the performance of most standard classification methods [6]. Because minor classes are ubiquitous in remote sensing, the data sets are often imbalanced. In general, there are three major ways to cope with imbalanced data sets: (i) to adapt the classification algorithm to reinforce learning of the minor classes, (ii) to adjust the classifier by assigning different costs to misclassification in rare versus frequent classes or (iii) resample the data set (oversampling and undersampling) (see [6] and references therein). This last approach has the advantage of being independent of the classifier used.

Oversampling of the rare classes with replacement or undersampling of the major class have been discussed by several authors (e.g., [7]). However, the potential of these approaches to improve the classification accuracy of rare classes seems to be limited. In particular random oversampling with replacement can lead to overfitting. To overcome this issue, Chawla et al. [8] proposed to generate new minority instances by a synthetic minority oversampling technique (smote). They reported that the synthetic points created by smote forced the classifier to learn larger and less specific regions which leads to a better performance than oversampling the minority class by replacement. The original smote algorithm was extended to prevent possible overgeneralization and class overlap [9].

In remote sensing, Random Forests (rf) have been widely adopted for lulc classification (e.g., [10]). While many more different classifiers exist, it has been reported that rf performed best over a wide variety of data sets (121 data sets, with rf surpassing 90% of the maximum accuracy in 84% of the data sets) [11] and we therefore combine rf with smote. Indeed, recent studies have demonstrated the usefulness of smote for remote sensing applications [12, 13].

Nowadays, most of the agricultural land is used for intensive food production. Depending on agricultural practices the intensification often comes at the cost of land degradation (e.g., [14]). Thus, agricultural areas often exhibit a typical conflict between ecosystem services, namely between provisioning services (i.e. crop production) and regulating services (e.g. erosion prevention and maintenance of soil fertility). In particular, under monsoon climate, soil erosion constitutes a major environmental issue [15]. In mountainous agricultural regions in South Korea, for example, annual dryland crops are often cultivated on slopes. The combination of steep slopes, low soil cover and heavy rainfalls increases the risk of soil erosion [16, 17]. In contrast to annual crops, perennial crops can provide soil cover during the whole year and help to prevent soil loss. Therefore, the shift from annual to perennial crops is encouraged by policy measures.

In our work, we use smote to improve the classification of annual and perennial crops in a mountainous agricultural catchment. This data set is characterized by a large imbalance ratio. It contains 6 classes, a major one covering more than 50% of the area and 5 minor classes. The goal of our study is to improve the classification of the rare classes ‘annual dryland crops’ and ‘perennial crops’ by using rf with smote on modis time series. We compare four different classification scenarios on original imbalanced and synthetically oversampled data. We quantify the effect of smote on overall classification performance and on different groups of land cover classes. In particular, we show that in the presence of class overlap, synthetically increasing the number of training points using smote does not guarantee a better classification result. To our knowledge, only few papers in the remote sensing literature address this issue on a complex real-world data set with imbalanced classes. Finally, we analyse by which mechanism the alternative scenarios affect model performance, looking at the relationship between class labels and surface reflectance (mutual information) and the difficulty of classification (entropy).

## Materials and methods

### Study area

**Fig 1:**
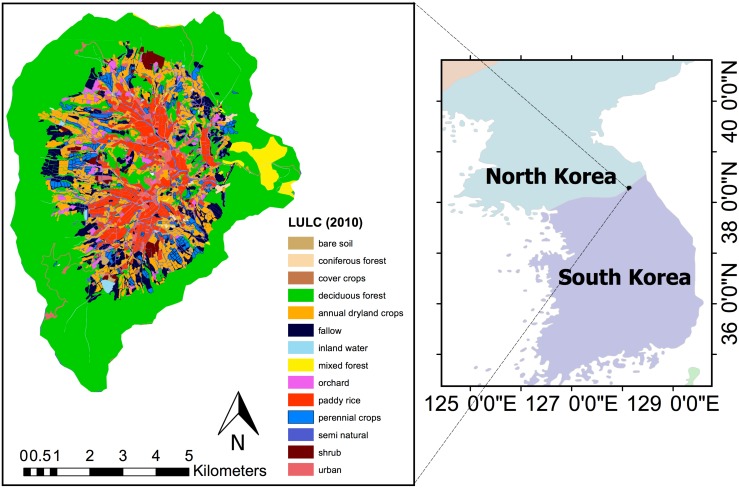
Land use and land cover and location of the Haean catchment. The map on the left shows the original polygon data set by [20] (WGS84 / UTM 52N; EPSG:32652) for the year 2010 with the 14 lulc classes used in this study.

The studied catchment Haean (128°1′33.101′′E, 38°28′6.231′′N) is situated in the mountainous watershed Soyang in the northeastern part of South Korea (Fig 1). It has a total area of 64 km^2^ with elevations ranging from 500 m to 1200 m. The agricultural zone is located in the center of the catchment and has a mosaic structure with paddy field rice as dominant crop and various annual dryland crops grown on the slopes. The catchment is surrounded by a dense deciduous forest.

Haean belongs to the East Asian summer monsoon region with heavy rainfalls occurring from June to August. The annual average rainfall equals 1599 mm and the maximum daily rainfall was 223 mm between 1999 and 2010 (Korean Meteorological Administration, http://web.kma.go.kr/eng, accessed on 2017-11-01). More than 60% of the annual precipitation is concentrated in the monsoon season with frequently occurring extreme events. Therefore, the acquisition of optical spectral data during summer is generally difficult and satellites with long revisit times like landsat rarely provide cloud-free time series during the growing season.

Intensive agriculture combined with erodible soils on steep slopes has transformed the catchment into a major source of pollution of the North Han River that supplies several cities (including the capital Seoul) with drinking water [18, 19]. The current agricultural practice to grow annual dryland crops in ridges with plastic cover enhances soil erosion [16]. Accordingly, a series of policy measures was initiated by the local government. In particular, the conversion of annual crops grown on hillslopes to perennial crops like ginseng is being subsidized and causes rapid changes in lulc [20]. A previous study showed how crop selection affected farmers’ revenue [21]. Thus, conversion of annual to perennial crops could have an impact on local economy. lulc maps that contain accurate information on minor classes like ‘annual dryland crops’ and ‘perennial crops’ are important to track land use changes and assess the success of the policy measures.

### MODIS surface reflectance

modis has a daily revisiting time and the data can be temporally aggregated to mitigate gaps (e.g. due to cloud contamination). We used the Vegetation Indices product MOD13Q1 (Collection 6) that has a spatial resolution of 250 m and provides four reflectance bands (bands 1, 2, 3 and 7). They contain specific information related to their spectral ranges. The red band 1 (B1) covers 620–670 nm and is sensitive to chlorophyll in vegetation. The near-infrared band 2 (B2) covers 841–876 nm and has been widely used to study vegetation together with B1. Band 3 (B3) is commonly called the blue band due to its sensitivity to water vapour. It covers 459–479 nm and is used to filter the cloud covered data or detect water bodies. Additionally, it serves to differentiate soil from vegetation. The range of the mid-infrared band 7 (B7) equals 2105–2155 nm and is also used to examine land and cloud properties (LP DAAC, https://lpdaac.usgs.gov/dataset_discovery/modis, accessed on 2017-11-01). Note that the channels B3 and B7 are originally acquired at 500 m resolution, but delivered at 250 m resolution in MOD13Q1 after downscaling done by NASA [22].

Every observation in MOD13Q1 is a temporal composite of a 16-day measurement period [23]. We used the data for the year 2010. Every band contained 23 images (i.e. 23 acquisition dates) and we obtained 92 data points per pixel of 250 m × 250 m. The whole catchment covered 1200 pixels of the modis tile H28V5. In the same study area, Seo and colleagues [24] used reflectance bands together with vegetation indices to predict fractional lulc cover. However, they based their study on a modis product with a lower spatial resolution (500 m). In order to restrain the number of predictors and decrease the computation time we refrained from including the vegetation indices.

Although MOD13Q1 already consists of the best measurements acquired during every 16 day composition period, it still contains a substantial amount of cloud contamination in our research area. This is largely due to the monsoon climate and therefore frequent cloudy weather during the vegetation period. Additionally, snow and ice are common during winter in mountainous areas. Therefore, we excluded images that were largely cloud or ice contaminated, namely the first four images (acquisition dates 2010-01-01 to 2010-02-28) and the last image (acquisition date 2010-12-20 to 2011-01-03). From the remaining 18 images, we removed all pixels that had three or more consecutive cloud contaminated acquisition dates (30 pixels) and four or more cloud contaminated acquisition dates during summer (54 pixels, acquisition dates 2010-05-09 to 2010-08-29). The remaining 1116 pixels contained each a time series of 18 values. These time series were filtered in four steps.

1. Removing spikes in bands 1, 3 and 7: Cloud contamination during summer was visible in bands 1, 3 and 7 by large positive values. Although MOD13Q1 provides quality flags, they do not always capture residual cloud contamination. In the first step we set all acquisition dates to NA that contained values larger than the 75% quantile of the respective time series in summer (acquisition dates 2010-05-09 to 2010-08-29) regardless of the quality flags. Because reflectance is usually larger in band 2, the cloud contamination was less obvious and we did not remove any values in this band. The bands 1, 3 and 7 were processed individually.
2. Closing gaps: Subsequently, we closed the gaps that were already present in the original data or introduced in step 1 by loess (locally weighted regression, degree = 2, span = 0.5, [25]) with weights derived from the quality flags in the “VI usefulness” layer (S1 Table).
3. Median filtering: This gap-free data was then median filtered with a window width of 5 and all points with low quality were replaced by filtered data. We considered all data with quality flags equal to 3 in the “VI quality” layer and quality flags ≥ 5 in the “VI usefulness” layer as low quality.
4. Final smoothing: We smoothed the data by loess with the same weights as in step 2. The true acquisition dates differed between pixels in the study area (because MOD13Q1 is a 16 day composite product). Therefore, we calculated 18 evenly spaced time points between the minimum and maximum acquisition dates and predicted all time series for those dates.

In summary, the filtering provided 1116 gap-free smoothed times series of length 72 (i.e. at 18 common dates for four bands). The original and the filtered data are shown in the Supplementary Information for comparison (S1 and S2 Figs).

### Reference land use and land cover data

The reference lulc data set was obtained by ground census of the whole study area in 2010. It contains 67 crop/non-crop lulc classes and is available online at the public repository Pangaea [20]. The data set consists of projected geospatial polygons (WGS84 / UTM 52N; EPSG:32652) with lulc classes assigned to each polygon and with detailed information on grown crops. Because we were interested in improving the classification of ‘annual dryland crops’ and ‘perennial crops’ in general, we gathered different crops belonging to these groups together (S2 Table).

For each of the 1116 modis pixels covering the study area, we determined the lulc class using this polygon data set. If a pixel contained multiple polygons (e.g. several agricultural fields with different crops), we assigned the lulc class covering the largest proportion of the pixel. This procedure yielded a raster data set containing 14 lulc classes (Table 1).

**Table 1:** Distribution of the 14 LULC classes in the raster data set. The first 6 classes were used for classification.

Nr	lulc class	Pixels		Area	
		(#)	(km^2^)	(km^2^)	(%)
1	deciduous forest	691	57.58	35.67	55.41
2	annual dryland crops	179	14.92	7.34	11.40
3	paddy rice	135	11.25	5.18	8.04
4	fallow	82	6.83	4.55	7.07
5	perennial crops	35	2.92	1.95	3.03
6	mixed forest	22	1.83	1.34	2.08
7	urban	14	1.17	1.65	2.57
8	shrub	12	1.00	0.92	1.43
9	semi natural	11	0.92	3.85	5.98
10	orchard	11	0.92	0.95	1.48
11	inland water	3	0.25	0.56	0.86
12	bare soil	3	0.25	0.14	0.22
13	coniferous forest	2	0.17	0.19	0.29
14	cover crops	0	0	0.06	0.09
15	NA	0	0	0.02	0.03

### Spectral similarity between classes

Spectrally similar classes are difficult to discriminate. Similarity between classes is termed class overlap and is a major difficulty in classification (e.g., [26]). While smote, by construction, reduces the imbalance between major and minor lulc classes, it cannot eliminate a possible class overlap that already exists in the original data. To assess spectral similarities between lulc classes in the original (imbalanced) data set, we calculated the Jeffries–Matusita distance (*JMD*) [27]. *JMD* is a spectral separability measure that can be used to quantify similarities between classes and hence class overlap. It compares distances between distributions of classes (e.g., [28]). For normally distributed classes *C*_1_ and *C*_2_ it equals

$$\begin{array}{c} JMD\left(C_{1},C_{2}\right)=2\cdot (1-e^{-B}) \end{array}$$ (1)

with *B* being the Bhattacharyya distance

$$\begin{array}{c} B\left(C_{1},C_{2}\right)=\frac{1}{8}{\left(m_{1}-m_{2}\right)}^{t}{\left(\frac{\Sigma_{1}+\Sigma_{2}}{2}\right)}^{-1}\left(m_{1}-m_{2}\right)+\frac{1}{2}\text{ln}\left(\frac{\left|\left(\Sigma_{1}+\Sigma_{2}\right)/2\right|}{|\Sigma_{1}{|}^{1/2}{\left|\Sigma_{2}\right|}^{1/2}}\right) \end{array}$$ (2)

where *m*_1_ and *m*_2_ are the vectors of means of classes *C*_1_ and *C*_2_ and *Σ*_1_ and *Σ*_2_ their covariance matrices, respectively. The calculation of *JMD* requires the inversion of the covariance matrices estimated from the spectral data. In our case, the covariance matrices are singular for two lulc classes because the number of dimensions (72) exceeds the number of samples (Table 1). Therefore, we calculated *JMD* for every modis band separately. However, for the smallest class ‘mixed forest’ the covariance matrices for bands B3 and B7 were nevertheless singular and we used the Moore–Penrose inverse (a generalized inverse) instead of the ordinary one. For this class, *JMD* must be interpreted with caution as the calculation could have been affected by the ill-conditioning of the covariance matrix. *JMD* equals 0 for two identical distributions and approaches 2 asymptotically for two completely separable classes. Thus, pixels of classes *C*_1_ and *C*_2_ would be correctly classified into their respective classes with an accuracy of 100% for *JMD* = 2, provided the classification problem is binary (i.e. *C*_1_ and *C*_2_ are the only classes considered) [28].

### Resampling and preprocessing of training data

**Fig 2:**
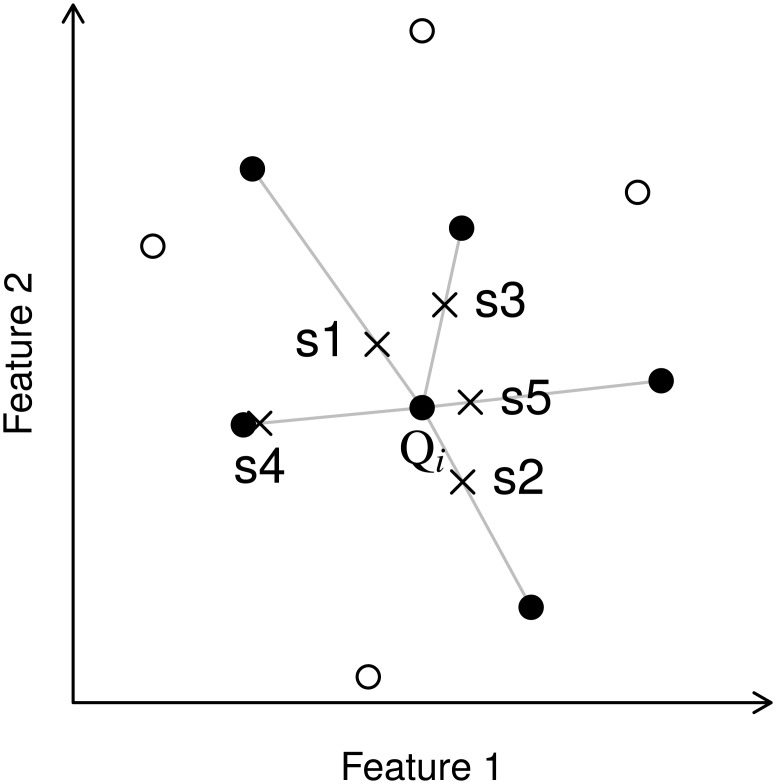
Illustration of SMOTE. Synthetic points (crosses denoted *s*1 through *s*5) generated by smote along the connection lines between a point (black dot denoted *Q*_*i*_) and its *k* nearest neighbours (black dots). Here, *k* = 5 and oversampling rate *N* = 5.

The synthetic minority oversampling technique (smote) oversamples a rare class by generating new instances [8]. For every existing point *Q*_*i*_ (in our case modis pixel) in a given rare class, it inserts synthetic points along a line that connects this point to one of its *k* nearest neighbors. Depending on the oversampling rate *N*, several *k* nearest neighbors can be chosen randomly and several points can be generated along one connecting line (Fig 2). In our case, smote can be understood intuitively as a linear spectral mixing method because it linearly combines two spectral signals (i.e. modis time series) to generate a new synthetic time series.

The original smote algorithm suffers from several drawbacks [6, 9, 29]. In particular, the information on the distribution of all other classes is ignored, which can lead to overgeneralization and implausible synthetic points located spectrally close to other classes. This increases class overlap and decreases the classification accuracy. Several methods were presented to tackle this problem, e.g. borderline-smote [30], safe-level-smote [29], and its variant ln-smote (Local-Neighborhood-smote) [9]. For our study, we have chosen the ln-smote algorithm because it is straightforward to understand and easy to implement.

The basic approach is identical to the original smote algorithm. A random point *Q*_*i*_ in a rare class *C*_*i*_ is chosen and the set *knn*(*Q*_*i*_) of the *k* nearest neighbors of *Q*_*i*_ is calculated. In contrast to the original smote algorithm, the neighbors do not mandatorily belong to the same class because the algorithm looks at actual neighborhood and not only at points of the same class. However, if *Q*_*i*_ and *Q*_*n*_ are from different classes, special care is taken when generating new synthetic samples (see details below). A random point *Q*_*n*_ is drawn from the set *knn*(*Q*_*i*_). The new synthetic instance lies on the line between *Q*_*i*_ and *Q*_*n*_ described by the vector ${\vec{Q}}_{in}={\vec{Q}}_{n}-{\vec{Q}}_{i}$. The synthetic sample is then calculated as

$$\begin{array}{c} n\vec{e}w={\vec{Q}}_{i}+\delta \cdot {\vec{Q}}_{in}\; \end{array}$$ (3)

where *δ* describes a weight term drawn from a uniform distribution *random*(*a*, *b*) with lower limit *a* and upper limit *b*. The idea of the procedure is to determine suitable values for *a* and *b* based on the spatial distribution of the samples. For this, the corresponding *k* nearest neighbors of *Q*_*n*_, *knn*(*Q*_*n*_), are selected. For both *Q*_*i*_ and *Q*_*n*_ the safe level is calculated. It is defined for a point *Q* as

$$\begin{array}{c} sl(Q):=|\{q:q\in knn(Q)\wedge class(q)=class(Q)\}|. \end{array}$$ (4)

The safe level of a point *Q* is the count of samples in *knn*(*Q*) that belong to the same class as *Q*. This measure is calculated for *Q*_*i*_ and *Q*_*n*_. Based on these, the safe level ratio *slr*: = *sl*(*Q*_*i*_)/*sl*(*Q*_*n*_) can be defined. Depending on its value, five different cases can be evaluated to determine appropriate values for *a* and *b*:

1. *sl*(*Q*_*i*_) = 0 ∧ *sl*(*Q*_*n*_) = 0: Both samples are surrounded only by samples from other classes, therefore they can be classified as noise and no synthetic instance is generated.
2. *sl*(*Q*_*i*_) > 0 ∧ *sl*(*Q*_*n*_) = 0: *Q*_*n*_ is surrounded solely by samples from other classes and can be classified as noise. Thus, the synthetic instance should be very close to *Q*_*i*_. Therefore, *a* and *b* are set to 0 and *Q*_*i*_ is duplicated.
3. *slr* = 1: The neighborhood of *Q*_*i*_ and *Q*_*n*_ is identical regarding the safe-level. The same approach as in the original smote is used, so *a*: = 0 and *b*: = 1.
4. *slr* > 1: The neighborhood of *Q*_*i*_ is more suited for a synthetic instance, so it should be closer to *Q*_*i*_. This can be achieved by *a*: = 0 and *b*: = 1/*slr*.
5. *slr* < 1: This is analogous to case 4, therefore *a*: = 1 − *slr* and *b*: = 1

Based on the parametrized distribution *random*(*a*, *b*) the weight term *δ* can be determined. If the classes of *Q*_*i*_ and *Q*_*n*_ differ, it is modified as *δ* ≔ *δ* ⋅ *sl*(*Q*_*n*_)/*k*, to bias the new synthetic instance towards the same class as *Q*_*i*_. Analogous to smote, this procedure can be repeated accordingly to a given oversampling ratio *N*.

The critical parameter in this algorithm is the number of nearest neighbors *k*. On the one hand, if it is too large, it has a high impact on class overlap and other possible errors (e.g. from subclasses inside a class). On the other hand, if *k* is too small, samples could be misclassified as noise because their safe ratio could be zero. Following the suggestions in previous studies [8, 9] we used *k* = 5.

In order to generate synthetic data, the rare class must contain at least some original points. The distribution of the lulc classes in the Haean catchment is highly imbalanced and dominated by ‘deciduous forest’. For smote we selected the 6 largest classes that contained at least 20 pixels (Table 1). Together they make up 95% of the total number of pixels and 87% of the total area of the catchment.

Additionally to oversampling the minority classes, we removed Tomek links by undersampling the majority class ‘deciduous forest’ in order to reduce the overlap with the smaller classes [31]. Tomek links are direct neighbors belonging to different classes and are either borderline instances or one of them is noisy.

### Mutual information: Relationship between class labels and surface reflectance

Changing the distribution of classes by data resampling might affect the relationship between the lulc class labels and modis spectral bands. Spectral bands related to minor classes, for example, could become more influential. To quantify this change we calculated the mutual information *MI*—a general measure of dependency between random variables

$$\begin{array}{c} MI(X,Y)=H(X)+H(Y)-H(X,Y)\;, \end{array}$$ (5)

where *H*(*X*) and *H*(*Y*) are the Shannon entropies of the random variables *X* and *Y*, respectively, and *H*(*X*, *Y*) is the joint Shannon entropy of *X* and *Y* [32]. We estimated *MI* by the method proposed by Kraskov and colleagues [33].

The mutual information is non-negative, but has no upper bound. In order to facilitate comparisons between different data sets, we normalized it [34]:

$$\begin{array}{c} MI^{*}\left(X,Y\right)=\text{sign}\left(\hat{MI}\left(X,Y\right)\right){\left(1-e^{-2\left|\hat{MI}\left(X,Y\right)\right|}\right)}^{\frac{1}{2}}, \end{array}$$ (6)

where $\hat{MI}(X,Y)$ is the estimate of the mutual information and the function sign(⋅) evaluates the sign of its argument. Although *MI* is non-negative by definition, its estimate $\hat{MI}$ could be negative indicating an estimation error. If $\hat{MI}(X,Y)\geq 0$, then MI*(*X*, *Y*) ∈ [0, 1]. MI*(*X*, *Y*) measures the overall dependency between *X* and *Y* providing an indicator of how well information from one can be used to reduce uncertainty of the other. It is 0 iff *X* contains no information about *Y* (i.e. *X* and *Y* are statistically independent), approaches 1 for increasing $\hat{MI}(X,Y)$ and equals 1 if there is a deterministic functional relationship between *X* and *Y*.

### Performance measures

Usually the performance of a classifier is assessed via the confusion matrix that shows the number of correctly classified positive (*TP*: True Positive) and negative examples (*TN*: True Negative) and misclassified positive (*FP*: False Positive) and negative examples (*FN*: False Negative). A classical measure of performance is the overall accuracy *A* = (*TP* + *TN*)/(*TP* + *FP* + *TN* + *FN*). However, with imbalanced data sets, *A* can be inappropriate because it masks a poor performance on minority classes [6]. To summarize the confusion matrix in our multi-class classification task, we calculated the macro-averaged *precision* (also known as user’s or consumer’s accuracy), *recall* (also known as producer’s accuracy), *F*-*score* and *G*-*mean*. We used the macro-averaging for multi-class problems because it treats all classes equally [35]. The macro-averaged precision is calculated as

$$\begin{array}{c} P_{M}=\frac{1}{M}\sum_{i=1}^{M}P_{i}\;, \end{array}$$ (7)

where *P*_*i*_ = *TP*_*i*_/(*TP*_*i*_ + *FP*_*i*_) is the precision in class *i* and *M* the number of classes. The macro-averaged recall is defined as

$$\begin{array}{c} R_{M}=\frac{1}{M}\sum_{i=1}^{M}R_{i} \end{array}$$ (8)

where *R*_*i*_ = *TP*_*i*_/(*TP*_*i*_ + *FN*_*i*_) is the recall in class *i* and *M* the number of classes. Precision is a measure of exactness and reports what fraction of the data labels of a class recognized by the classifier really belongs to the class. Recall shows the effectiveness or completeness of a classifier to find all the actually correct class labels [6]. *F*-*score* [36] is a combination of *precision* and *recall*.

$$\begin{array}{c} F\text{-}score=\frac{2P_{M}R_{M}}{P_{M}+R_{M}}\;. \end{array}$$ (9)

The multi-class version of *G*-*mean* [37] measures the balanced performance of a classifier and is defined as

$$\begin{array}{c} G-mean={\left(\prod_{i=1}^{M}R_{i}\right)}^{\frac{1}{M}}, \end{array}$$ (10)

where *R*_*i*_ is the recall in class *i* and *M* the number of classes.

As a general measure of difficulty of a classification task, we used the entropy. As pointed out by Kononenko and Bratko [38] the distribution of the classes is closely related to the difficulty of the classification task. Consider a classification task with *M* classes *C*_*i*_, *i* = 1, …, *M* with prior probabilities *p*(*C*_*i*_). We require ∑_*i*_ *p*(*C*_*i*_) = 1. The amount of information to correctly classify one instance with prior probability *p*(*C*_*i*_) into class *C*_*i*_ equals − log *p*(*C*_*i*_) and to correctly state that it does not belong to this class equals − log(1 − *p*(*C*_*i*_)). Then, the expected amount of information to classify one instance equals to the entropy

$$\begin{array}{c} H=-\sum_{i}^{M}p\left(C_{i}\right)\log p\left(C_{i}\right)\;. \end{array}$$ (11)

Thus a classification task with equal prior probabilities *p*(*C*_*i*_) = 1/*M* is the most difficult one and has the largest entropy (log *M*). A classifier trained on an unbalanced data set is more at risk to specialize on the majority class and to neglect minority classes. Actually, by classifying every instance into the majority class it can attain a high accuracy. Therefore, changing the distribution of data affects the difficulty of the classification task.

The agricultural area in the Haean catchment has a mosaic-like structure. Additionally to the performance metrics mentioned above, we wanted to assess how well this structure is reproduced in the classification scenarios. Therefore, we calculated the mean patch density (*PD*) for every class—a metric that characterizes the geometry of landscape patterns [39]:

$$\begin{array}{c} PD=\frac{n_{i}}{A} \end{array}$$ (12)

where *n*_*i*_ are the number of patches of a lulc class *C*_*i*_ and *A* the area of the catchment, respectively [40].

### Classification scenarios

In order to evaluate how the classification performance changes when the training data is preprocessed, we compared different classification scenarios. In every scenario, we carried out a 6-fold stratified cross validation (scv). Thus we split the data randomly (each class separately) in 6 folds, used 5 folds to train the classifier and the hold-out fold to test it. Because each fold was used once as a hold-out test fold, we obtained predictions for every pixel. The random splitting was repeated 10 times. Note that we always evaluated the scenarios on the hold-out test set that was not preprocessed (i.e. neither synthetically oversampled, nor undersampled nor Tomek links removed).

We used the same splitting in training and test data for all scenarios. These were defined as follows:

**S1: Original data** This is the base-line scenario. Any data resampling procedure and subsequent classification, if it is to have any potential utility, has to perform at least as well as in this scenario.
**S2: Original data with Tomek links removed** We removed Tomek links in the majority class ‘deciduous forest’ in the training data.
**S3: Smoted training data** The goal of this scenario is to obtain an approximately equal distribution of minority classes for training. Therefore, after removing Tomek links as in S2, the minority classes in the training folds were synthetically oversampled approximately up to the number of ‘annual dryland crops’ pixels (i.e. 145 pixels). The classes ‘deciduous forest’, ‘annual dryland crops’ and ‘paddy rice’ were not oversampled.
**S4: Smoted training data and undersampling** Additionally to the removal of Tomek links and smote as in S3, the majority class ‘deciduous forest’ was undersampled by randomly selecting 145 pixels for training (corresponding to the number of ‘annual dryland crops’ pixels in the training data) to obtain an approximately equal distribution of all classes in the training folds.

### Parameter tuning

rf is an ensemble technique that uses decision trees. For every decision tree a new bootstrap sample of the training data is created and the tree is fitted to the data. The final solution of the classification problem is the plurality vote over all decision trees. rf has three hyperparameters, namely the number of trees (*n*_*tree*_), the number of randomly selected predictors in each split of the decision tree (*m*_*try*_) and the minimum number of samples in terminal nodes (*nodesize*). Several studies have shown that the number of trees needs to be large for a good model performance and does not yield to overfitting [41, 42]. Following advice by Kuhn and Johnson [42], we set the hyperparameter *n*_*tree*_ to 1000. The hyperparameter *nodesize* was set to 1, a default value for classification (i.e. the single trees were not pruned but grown to their maximum size). By contrast, *m*_*try*_ should be tuned to avoid overfitting, in particular when predictors are correlated (e.g., Strobl2009, Kuhn2013 and references therein). We performed a grid search to find the optimal *m*_*try*_ per scenario on a grid from 2 to 72. This optimization was done in an internal 5-fold scv on the training folds only, using *F*-*score* as the optimization criterion.

All calculations in our study were done using R [43] and add-on packages (S1 Appendix).

## Results

### Spectral profiles and similarities between classes

**Fig 3:**
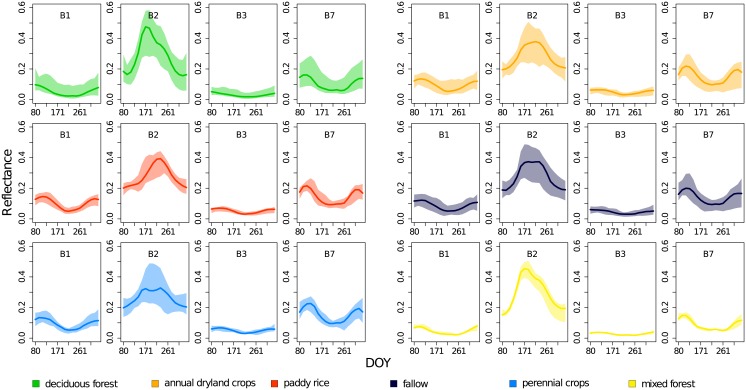
Spectral profiles. Reflectance values of all pixels in the original data set in the red band B1, the near-infrared band B2, the blue band B3 and the mid-infrared band B7. The plain lines show the median and the shaded areas the range (from minimum to maximum value). DOY is a day of the year derived from the true acquisition date through interpolation.

The spectral profiles (i.e. the modis time series) of the original data show similar phenological patterns (Fig 3) in the two forest classes ‘deciduous forest’ and ‘mixed forest’. The leaf flushing (identifiable by the increase in reflectance in the near-infrared channel B2) occurred earlier and quicker than in the agricultural lulc classes. In addition, the maximum of reflectance in B2 is reached earlier in the year (except in ‘fallow’). The large range between the minimum and the maximum values indicates a substantial spread and thus an intra-class variability of spectral profiles.

**Fig 4:**
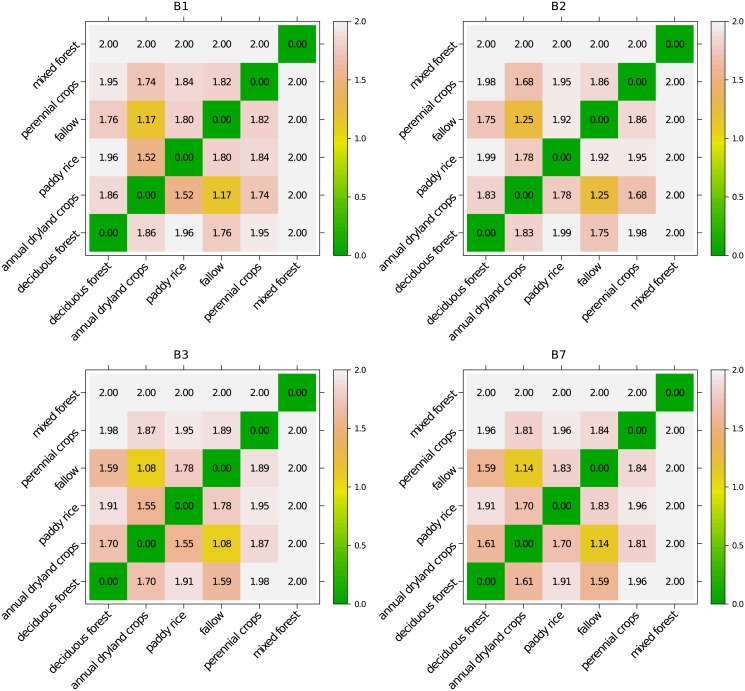
Jeffries–Matusita distances. Jeffries–Matusita distances between different classes in the original data calculated for every modis band.

The pairwise Jeffries–Matusita distances ranged between 1.08 and 2 (Fig 4). We expect the strongest confusion between ‘fallow’ and ‘annual dryland crops’ with the smallest *JMD*. In contrast, *JMD* between the forest classes and the agricultural classes were large suggesting that they were separable. In particular, ‘mixed forest’ seemed to be particularly easy to classify. Hence, we expect a better separability between the forests and the agricultural classes than between the agricultural classes themselves. The differences between the *JMD* values between bands were small.

### Distribution of training data and SMOTE oversampling rate

The scenario S1 (original data set) was characterized by imbalance ratios ranging between 4: 1 (‘deciduous forest’ and ‘annual dryland crops’) and 31: 1 (‘deciduous forest’ and ‘perennial crops’) (Table 1). Because only 46 points (average value in the 10 repetitions) were identified as Tomek links and removed, the imbalance ratio in S2 remained comparable to S1. By contrast, beside the dominance of ‘deciduous forest’, the classes became evenly distributed in S3 after synthetic oversampling. Finally, the combination of synthetic oversampling of minority classes and random undersampling of the majority class ‘deciduous forest’ generated a nearly equal distribution of classes in S4. Note that Tomek links were also removed in S3 and S4.

The nominal synthetic oversampling rate *N* required to obtain an equal distribution for training pixels equaled 100% for ‘fallow’, 383% for ‘perennial crops’ and 667% for ‘mixed forest’. By contrast, the actual oversampling rate used by ln-smote was 76% for ‘fallow’, 276% for ‘perennial crops’ and 622% for ‘mixed forest’. This shows that some pixels were surrounded by neighbors from a different class and were ignored for oversampling. On average 63 new pixels were generated for ‘fallow’, while 97 and 137 new instances were created in the classes ‘perennial crops’ and ‘mixed forest’, respectively.

In their work, Chawla and colleagues [8] recommended to choose the oversampling rate not larger than the number of nearest neighbours *k*. For *k* = 5, for example, the oversampling rate should not exceed 500%. However, another study has shown that best classification results on data sets with a high imbalance ratio could be achieved with *N* four to five times larger than *k* [9]. In our work, we have chosen *k* = 5, thus our oversampling rates are large but likely not excessive.

### Difficulty of classification and relationship between class labels and surface reflectance

The classification problem on the original data was simpler compared to the other scenarios. Indeed, S1 had the lowest entropy (1.8) because the distribution of lulc classes was dominated by ‘deciduous forest’. Thus, the classifier could obtain a high overall accuracy (0.93, S4 Table) by classifying the majority classes correctly and performing worse on the minority classes. smote increased the difficulty of the classification in scenarios S3 and S4 (the entropy equaled 2.3 and 2.6, respectively) by balancing the distribution. In comparison, the maximum entropy for a classification problem of 6 classes equals log_2_6 = 2.6. Thus, the classification task in S4 attained the maximum difficulty.

**Fig 5:**
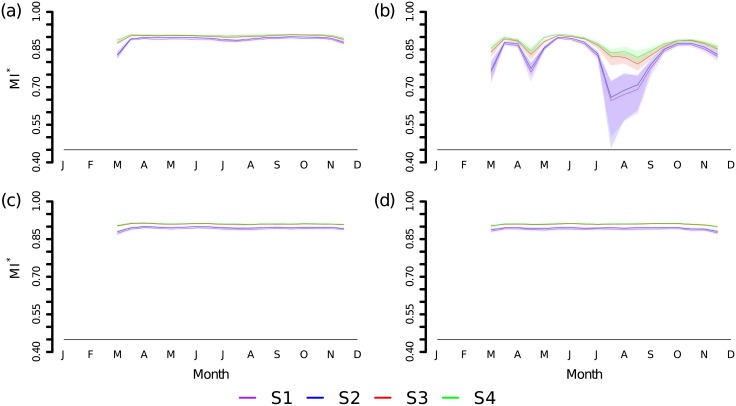
Mutual information MI* between class labels and MODIS spectral bands. Results from 10 repetitions on 6 training folds in scenarios S1 through S4: (a) red band B1, (b) near-infrared band B2, (c) blue channel B3 and (d) mid-infrared band B7. The plain lines show the median and the shaded areas the 5% to 95% quantile range.

The mutual information MI* between the surface reflectance and the class labels in the training data of scenario S1 was relatively large. However, it varied between the four modis bands (Fig 5). Especially in spring and late summer, MI* decreased in the near-infrared channel B2 that is sensitive to vegetation. Additionally, these periods showed the largest variability between the different runs in S1 and S2 and had therefore the broadest 5% to 95% quantile ranges.

The removal of Tomek links in S2 increased MI* in B2 only slightly without affecting its temporal shape. By contrast, compared to the original data, smote raised it noticeably and decreased its temporal variability in scenarios S3 and S4. Additionally, random undersampling of the majority class ‘deciduous forest’ in S4 further increased the mutual information.

### Classification performance

#### Overall performance of scenarios

The median *F*-*score* based on the 10 repetitions raised slightly from S1 (0.59) to S4 (0.63) (S5 Table). By contrast, *G*-*mean* increased substantially from 0.45 in S1 to 0.66 in S4. Despite only a small increase in *F*-*score*, *P*_*M*_ and *R*_*M*_ were affected by smote. Actually, *P*_*M*_ decreased from 0.66 in scenarios without oversampling (mean value for S1 and S2) to 0.60 in scenarios with smote (mean value for S3 and S4). On the other hand, *R*_*M*_ increased from 0.55 to 0.65. Thus, in scenarios with oversampling the rf classifier found more false positives. However, it also identified more actually positive instances and its performance was more balanced.

#### Classification of single LULC classes

**Fig 6:**
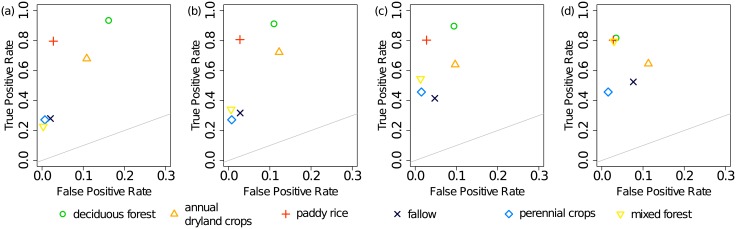
Classification results. Median values from 10 repetitions in scenarios S1 (a) through S4 (d). A point on the diagonal (grey line) indicates a random guess. The order of the classes in the legend reflects the decreasing number of original pixels.

Fig 6 summarizes the classification results. The false positive rate *FPR* = *FP*/(*FP* + *TN*) is plotted on the *x*-axis and the true positive rate *TPR* = *TP*/(*TP* + *FN*) on the *y*-axis. The more a point approaches (0, 1) in this graph, the better the performance of the classifier on this class.

In S1, the median *TPR*s of the majority class ‘deciduous forest’ was largest and equaled 94% (Fig 6a). Because many minority pixels were falsely classified as ‘deciduous forest’ the median *FPR* reached 16%. In particular, ‘annual dryland crops’, ‘fallow’ and ‘mixed forest’ were confounded with it (S3 Table). The classes ‘annual dryland crops’ and ‘paddy rice’ were reasonably well classified (TPRs 68% and 80%, respectively). Yet, the performance of rf on the minority class ‘perennial crops’ was low with a *TPR* of 27%. This class was mostly confounded with ‘annual dryland crops’.

In S2 the removal of Tomek links in the majority class ‘deciduous forest’ decreased its median *FPR* by 5%. Notably, less ‘annual dryland crops’ pixels were misclassified as ‘deciduous forest’ (S3 Table). Its *TPR* decreased by 2%. By contrast, it affected the *FPR*s in the other classes only slightly. Additionally, the median *TPR*s of all minority classes but ‘perennial crops’ increased (Fig 6b).

**Fig 7:**
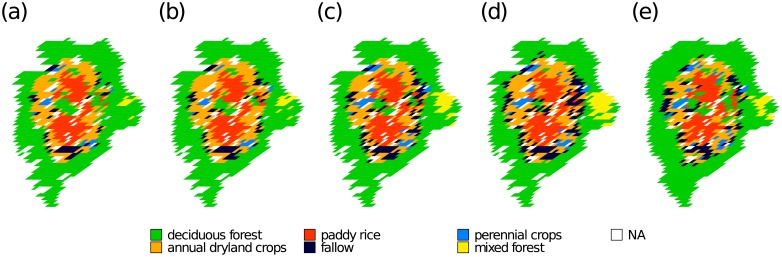
Predicted land use and land cover classes. Scenarios S1 (a) through S4 (d) and the original data set (e). The maps (WGS84 / UTM 52N; EPSG:32652) are from repetitions with the largest *F*-*score*. Classes with less than 20 original pixels and cloud contaminated pixels are marked as ‘NA’.

In both scenarios without oversampling, we observed a positive relationship between the median *TPR* of a class and the number of training pixels in that class. The Spearman’s rank correlation coefficient equaled 0.87 in S1 and 0.82 in S2. Additionally, minority classes at the boundary between ‘deciduous forest’ and the agricultural area as well as in the center of the catchment were underrepresented (Fig 7a and 7b. The classification results in S1 and S2 are in disagreement with *JMD* which indicated a good separability between ‘deciduous forest’ and the agricultural classes (Fig 4). Additionally, minor classes ‘perennial crops’ and ‘mixed forest’ with a large *JMD*s were misclassified.

The synthetic oversampling in S3 substantially increased the median *TPR*s in the minority classes ‘fallow’, ‘perennial crops’ and ‘mixed forest’ (Fig 6c). However, the median *TPR*s decreased for paddy rice (by <1%) and ‘annual dryland crops’ (by 8%). The pixels from the latter class were more frequently confounded with ‘fallow’ (S3 Table).

Finally, due to additional random undersampling of ‘deciduous forest’ in S4 the *TPR*s of the minority classes ‘mixed forest’ and ‘fallow’ increased compared to S3 (Fig 6d). However, the median *FPR*s also increased slightly as some ‘deciduous forest’ pixels were misclassified as ‘mixed forest’ or ‘fallow’ (S3 Table). Although the number of ‘deciduous forest’ pixels was reduced substantially, its median *TPR* decreased only by 8% (from 90% to 82%) and its *FPR* dropped from 9% to 3%.

In summary, smote decreased the *TPR*s of the majority class ‘deciduous forest’ and minority classes ‘annual dryland crops’ and ‘paddy rice’ only slightly. However, it substantially increased the *TPR*s of the minority classes ‘fallow’ ‘perennial crops’ and ‘mixed forest’. The correlation between the size of a class (i.e. number of training pixels) and *TPR* dropped (0.54 in S3 and 0.48 in S4). Additionally, the results agree with *JMD*, namely a good separability between ‘deciduous forest’ and the agricultural classes, confusion between ‘fallow’ and ‘annual dryland crops’ and ‘annual dryland crops’ and ‘paddy rice’, respectively. This also explains the relatively large *FPR* of the classes ‘annual dryland crops’ and ‘fallow’. The fragmented nature of the agricultural area appeared clearer (Fig 7c and 7d). In particular, the patch density of ‘annual dryland crops’ (S3: 25 per 100 ha, S4: 23 per 100 ha) and ‘perennial crops’ (S3: 12 per 100 ha, S4: 16 per 100 ha) increased and approached the density of the original data (‘annual dryland crops’: 30 per 100 ha and ‘perennial crops’: 23 per 100 ha) (S4 Fig).

## Discussion

### Influence of data resampling on classification performance of minority classes

Although *JMD* values indicated a potentially good separability between forest classes and agricultural classes and between some of the agricultural classes, the classification performance on minor classes in S1 and S2 remained actually low. This indicates that the number of training points was insufficient to train the classifier. Oversampling the training data decreased the imbalance ratio, and the classification performance in S3 and S4 was more in agreement with the *JMD* values. We attribute the misclassification of classes with large *JMD* values (e.g. ‘mixed forest’) to our multi-class setting. Indeed, as noted by Richards and Jia [28] a *JMD* value of 2 implies a perfect classification for a binary problem only. Additionally, *JMD* of ‘mixed forest’ could have been affected by the ill-conditioning of the covariance matrix (c.f. section Spectral Similarity Between Classes).

The positive relationship between the number of training pixels (i.e. the size of a class) and the ability of the classifier to recognize a pixel’s class correctly decreased in scenarios with oversampling. smote increased the difficulty of the classification task by balancing the distribution. Thus, the rf classifier attained a slightly larger *F*-*score* on a more complex problem. Additionally, smote raised the mutual information MI* between the lulc classes and the predictors, in particular in band B2. Thus, reflectances in the growing period became more relevant in data sets augmented by synthetic oversampling than in the original data set.

Both kinds of undersampling—removing Tomek links in S2 and random undersampling in S4—decreased the *FPR* and the *TPR* of the majority class ‘deciduous forest’. However, the relative decrease of the *TPR*, which did not fall below 80%, was smaller. It is quite obvious that removing Tomek links cleaned noisy pixels in ‘deciduous forest’. Random undersampling balanced the distribution of training data additionally to synthetic oversampling.

Overall, in scenarios S3 and S4 the classification of the oversampled class ‘perennial crops’ improved substantially (larger *TPR*s) and of ‘annual dryland crops’ remained satisfactory. The performance on the majority class ‘deciduous forest’ remained quite stable (*TPR*) or improved (smaller *FPR*).

### Class overlap—Major reason for misclassification

**Fig 8:**
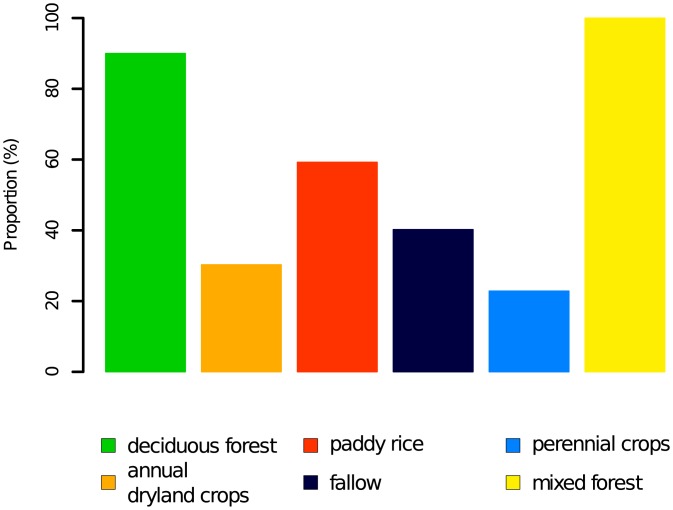
Composition of pixels. Proportion of pixels with more than 50% of the main lulc class in the original data set.

Although smote decreased the imbalance ratio and increased the mutual information between the lulc classes and the predictors, the classifier still performed best on the classes ‘deciduous forest’ and ‘paddy rice’. To better understand why minority classes were difficult to classify, we evaluated the fractional composition of the pixels (i.e. the proportions of different lulc classes) (Fig 8). Pixels with assigned classes ‘deciduous forest’, ‘mixed forest’ and ‘paddy rice’ are indeed dominated by those lulcs. A large proportion of these pixels contain more than 50% of the assigned class. By contrast, pixels with ‘annual dryland crops’, ‘fallow’ and ‘perennial crops’ are largely mixed with other classes. Such pixels are particularly difficult to classify because they overlap with other classes. smote increased the number of training points. However, classes like ‘fallow’ and ‘perennial crops’ are inherently mixed ones. Accordingly, the classifier performance remains low on these classes.

Our findings on the difficulty of classification related to class overlap are in agreement with previously published results. It has been widely accepted that class imbalance is often responsible for a bias towards dominant classes and the disagreement between large *JMD* values and low performance on minor classes in S1 and S2 confirms it. However, several recent studies report that imbalance *per se* does not prevent learning [26]. It is rather a combination of imbalance and intrinsic characteristics of the data like small sample size, possible sub-concepts (i.e. different clusters within single classes) or noisy data that all tend to increase class overlap. Indeed, because classification is basically a separation of distributions, any factor that makes distributions of different classes more equal (i.e. increases class overlap) could decrease the classification performance.

Therefore, class overlap plays a particular role in classification problems. Prati and colleagues [44], for example, reported that class overlap was at least as important as imbalance. They have shown on a set of artificial data that for the same imbalance ratio the performance of the classifier decreased with increasing class overlap. Moreover, other authors [45] described an interaction between overlap and imbalance and mentioned that for a certain degree of overlap, increasing the number of training samples did not improve the classification performance. To our knowledge, there are only few studies in remote sensing explicitly treating class overlap in particular in combination with data resampling. One of them is the work by Alejo and colleagues [46]. They also reported that smote failed to increase the classification performance in the presence of class overlap.

In our data set the interaction between overlap and imbalance can be observed when comparing the performance of the classifier on ‘perennial crops’ versus ‘mixed forest’. The number of training pixels in both lulc classes is comparable (Table 1). However, while ‘mixed forest’ consists of sufficiently many unmixed pixels, ‘perennial crops’ is a mixed class and increasing the number of its pixels does not guarantee a good classification result.

Overlap of lulc classes originates from spectral similarities between classes which can be enhanced by mixture of different crops inside the same pixel. In many agriculture mosaic landscapes, mixed pixels containing several crops are common because the majority of temporally rich and freely accessible satellite products have a coarse resolution. Therefore, pure pixels that reflect the spectral signature of one particular crop are often relatively scarce.

We are aware of the spatial and temporal limitations of our study. Indeed, the Haean catchment is relatively small, yet, a rather typical example for a mountainous ecosystem used for agricultural production in South Korea. The perennial shift (i.e. the replacement of annual by perennial crops) in the agricultural practices in the study area has started only recently and needs to be monitored in the future. However, the analysis of the temporal changes in lulc is beyond the scope of this work.

## Summary and conclusions

Our research was motivated by environmental issues like soil degradation and erosion associated with dryland agriculture on slopes practices in the study area. Shifting from annual to perennial crops is one of the mitigating measures. Therefore, our goal was to improve the classification of the classes ‘annual dryland crops’ and ‘perennial crops’ based on freely available remote sensing data in order to improve the monitoring of lulc changes.

In this study, we used smote to resample modis time series to decrease the imbalance ratio of the original data set. The classification of the original data was particularly challenging due to a small number of training points in the minority classes and the class overlap. smote helped to alleviate the issue of class imbalance by increasing the number of training points. The true positive rates of all oversampled minority classes raised substantially compared to the original imbalanced data set. However, due to class overlap the performance of the classifier on minority classes remained lower than on the majority class ‘deciduous forest’.

When oversampling fails to increase the classification performance, a detailed analysis of the fractional composition of pixels can yield important information. In particular, in the presence of class overlap, increasing the number of training points does not guarantee a better classification result. If there is some flexibility in the definition of classes, we recommend to perform an analysis of spectral separability in order to inform the definition of classes to improve the classification result.

Data preprocessing with smote to balance the data distribution is independent of the classifier. The implementation of the algorithm is straightforward and its functioning is easy to understand. We have opted for rf; however, any other classifier could be used on the preprocessed data. Therefore, synthetic oversampling can be plugged in into an existing classification framework without further adjustments.

In this study, we only used time series of one year and analysing changes over time was beyond the scope of this work. Although the temporal and spatial extent of the study are limited, it shows that preprocessing of data could improve lulc maps in heterogeneous agricultural areas and potentially benefit decision making.

## Supporting information

S1 Fig Original spectral profiles. The day of composite is the last day of a 16-day measurement period.
(PDF)

S2 Fig Spectral profiles after filtering. DOY is a day of the year derived from the true acquisition date through interpolation.
(PDF)

S3 Fig Distribution of training data in scenarios S1 through S4. (PDF)

S4 Fig Patch density in the original data and in scenarios S1 through S4. (PDF)

S1 Table Weights derived from the quality flags in the “VI usefulness” layer of MOD13Q1. (PDF)

S2 Table Reclassification scheme for the original 67 LULC classes. The 67 classes were combined to 14 classes and 6 classes containing more than 20 pixels were used in the study.
(PDF)

S3 Table Mean confusion matrices in 10 repetitions in scenarios S1 through S4. (PDF)

S4 Table Evaluation of the maps with the largest *F*-*score* in scenarios S1 through S4. (PDF)

S5 Table *F*-*score*, *G*-*mean*, precision and recall in 10 repetitions of scenarios S1 through S4. (PDF)

S6 Table ROC summaries as median *TPR* and *FPR* in 10 repetitions of scenarios S1 through S4. (PDF)

S1 Appendix List of R packages. (PDF)
