# Supplementary figures and images for: Classification of rare land cover types: Distinguishing annual and perennial crops in an agricultural catchment in South Korea
Source: PLoS One. 2018 Jan 25;13(1):e0190476. doi: 10.1371/journal.pone.0190476 (PMC5784906; doi:10.1371/journal.pone.0190476)

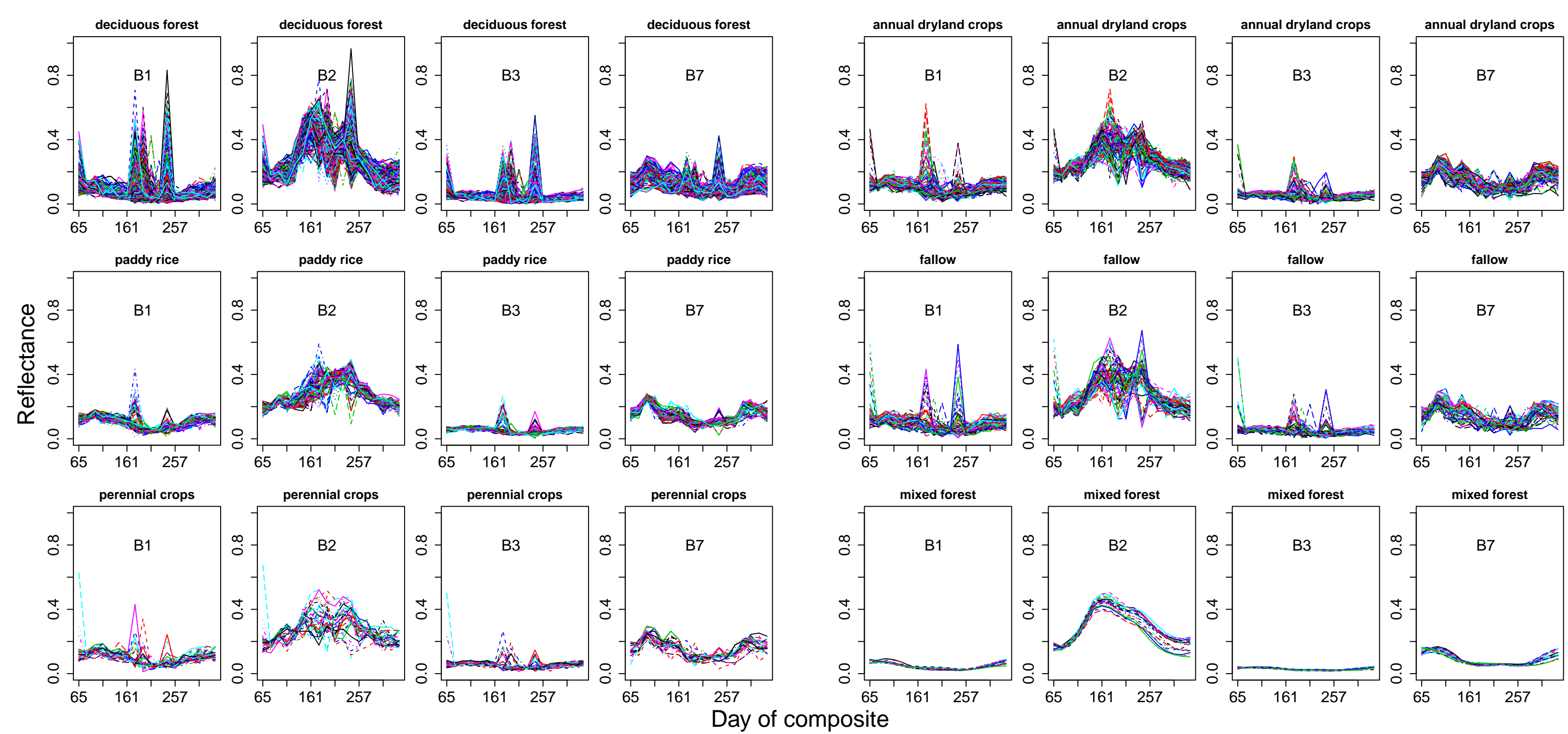

Supplement: S1 Fig — The day of composite is the last day of a 16-day measurement period. (PDF) [file pone.0190476.s001.pdf]

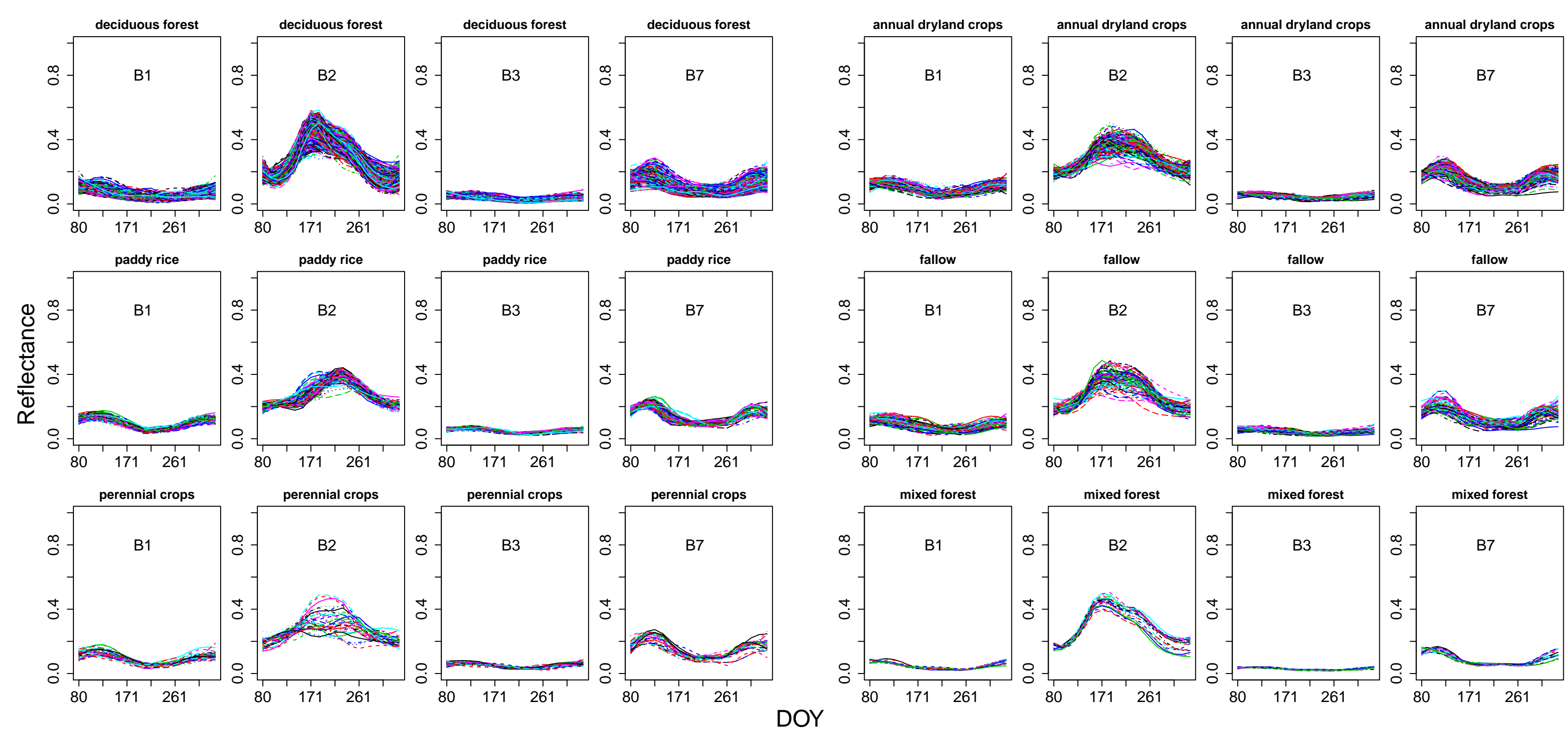

Supplement: S2 Fig — DOY is a day of the year derived from the true acquisition date through interpolation. (PDF) [file pone.0190476.s002.pdf]

Number of patches per 100 ha

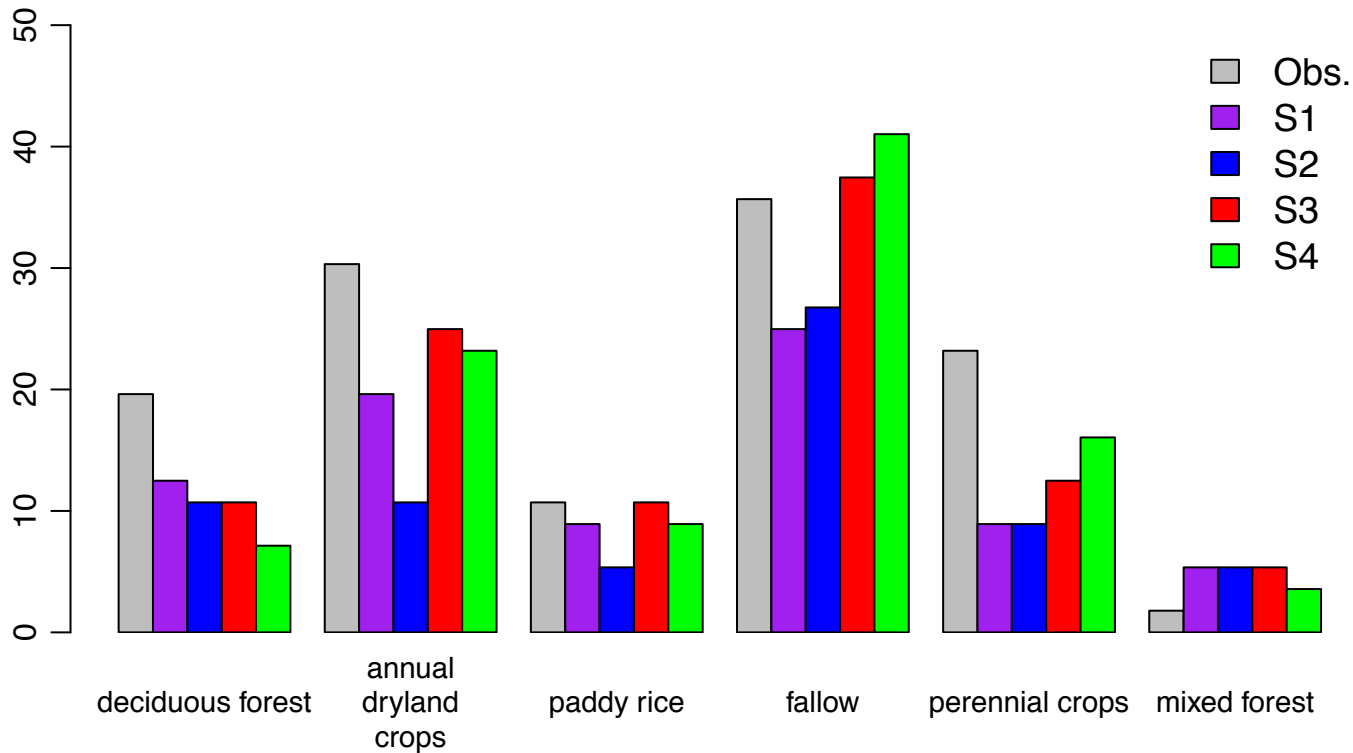

Supplement: S4 Fig — (PDF) [file pone.0190476.s004.pdf]
