# Supplementary material for: Classification of rare land cover types: Distinguishing annual and perennial crops in an agricultural catchment in South Korea
Source: PLoS One. 2018 Jan 25;13(1):e0190476. doi: 10.1371/journal.pone.0190476 (PMC5784906; doi:10.1371/journal.pone.0190476)
Supplement: S3 Fig — (PDF) [file pone.0190476.s003.pdf]

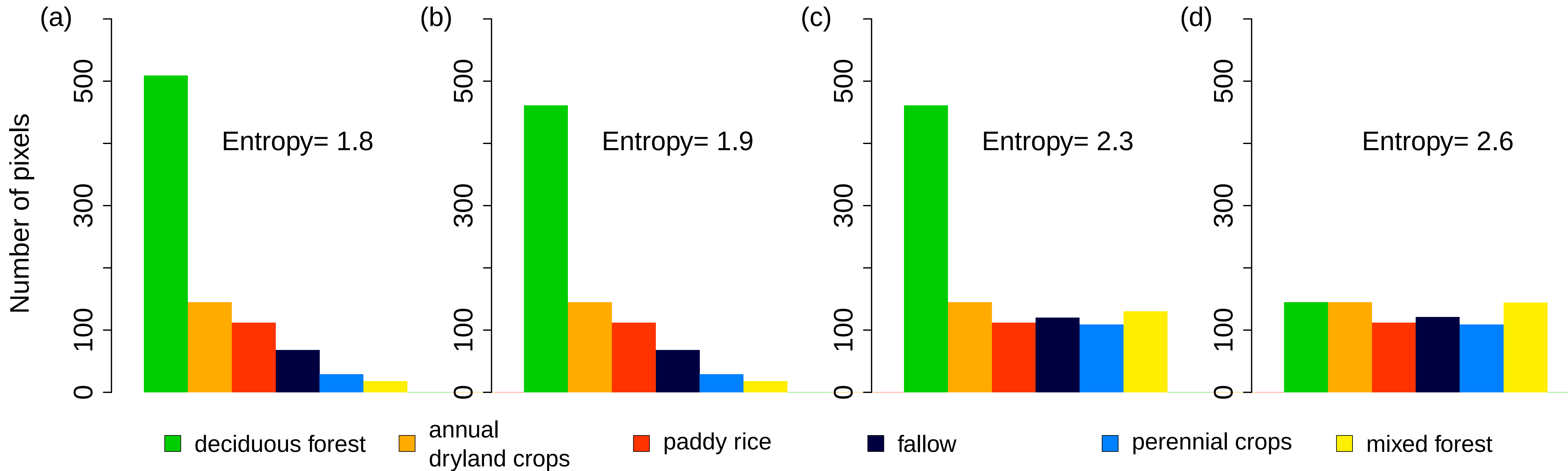

(a)
(b)
(c)
(d)
Number of pixels
0
100
300
500
Entropy= 1.8
Entropy= 1.9
Entropy= 2.3
Entropy= 2.6
deciduous forest
annual dryland crops
paddy rice
fallow
perennial crops
mixed forest
