# Supplementary material for: Classification of rare land cover types: Distinguishing annual and perennial crops in an agricultural catchment in South Korea
Source: PLoS One. 2018 Jan 25;13(1):e0190476. doi: 10.1371/journal.pone.0190476 (PMC5784906; doi:10.1371/journal.pone.0190476)
Supplement: S1 Table — (PDF) [file pone.0190476.s005.pdf]

VI usefulness flag	Interpretation	Weight
0	highest quality	1
1	lower quality	0.8
2	decreasing quality	0.5
3	decreasing quality	0.4
4	decreasing quality	0.3
5	decreasing quality	0.2
6	decreasing quality	0.1
7	lowest quality	0.05
8	quality so low that it is not useful	0
9	L1B data faulty	0
10	Not useful for any other reason/not processed	0
