# Supplementary material for: Classification of rare land cover types: Distinguishing annual and perennial crops in an agricultural catchment in South Korea
Source: PLoS One. 2018 Jan 25;13(1):e0190476. doi: 10.1371/journal.pone.0190476 (PMC5784906; doi:10.1371/journal.pone.0190476)
Supplement: S2 Table — The 67 classes were combined to 14 classes and 6 classes containing more than 20 pixels were used in the study. (PDF) [file pone.0190476.s006.pdf]

Original class	Reclassified class
deciduous forest	deciduous forest
maize, potato, bean, chinese cabbage, european cabbage, white radish, pepper, green pea, sesame, chicory, wrapping vegetables, fatsia pumpkin, broccoli, zucchini, lettuce, medicinal herb, green onion	annual dryland crops
paddy rice	paddy rice
fallow, tall grass	fallow
ginseng, codonopsis, *Aster scaber*, chinese bellflower	perennial crops
mixed forest	mixed forest
