# Supplementary material for: Classification of rare land cover types: Distinguishing annual and perennial crops in an agricultural catchment in South Korea
Source: PLoS One. 2018 Jan 25;13(1):e0190476. doi: 10.1371/journal.pone.0190476 (PMC5784906; doi:10.1371/journal.pone.0190476)
Supplement: S3 Table — (PDF) [file pone.0190476.s007.pdf]

Scenario S1

observed classes	predicted classes: deciduous forest	annual dryland crops	paddy rice	fallow	perennial crops	mixed forest
deciduous forest	571	28	3	5	1	3
annual dryland crops	22	119	20	10	4	0
paddy rice	7	20	107	1	0	0
fallow	26	30	1	23	2	0
perennial crops	2	19	0	5	9	0
mixed forest	17	0	0	0	0	5

Scenario S2

observed classes	predicted classes: deciduous forest	annual dryland crops	paddy rice	fallow	perennial crops	mixed forest
deciduous forest	557	35	3	8	1	6
annual dryland crops	12	126	20	12	5	0
paddy rice	2	22	109	2	0	0
fallow	21	32	1	26	2	0
perennial crops	0	20	0	4	10	0
mixed forest	15	0	0	0	0	7

Scenario S3

observed classes	predicted classes deciduous forest	annual dryland crops	paddy rice	fallow	perennial crops	mixed forest
deciduous forest	548	29	4	13	2	14
annual dryland crops	10	112	20	22	10	0
paddy rice	2	20	108	5	0	0
fallow	20	24	1	34	3	0
perennial crops	0	14	0	5	16	0
mixed forest	10	0	0	0	0	12

Scenario S4

observed classes	predicted classes deciduous forest	annual dryland crops	paddy rice	fallow	perennial crops	mixed forest
deciduous forest	500	40	5	34	2	30
annual dryland crops	2	113	20	29	11	0
paddy rice	0	20	109	6	0	0
fallow	9	25	1	43	3	1
perennial crops	0	14	0	5	16	0
mixed forest	4	0	0	0	0	18
