# Supplementary material for: Classification of rare land cover types: Distinguishing annual and perennial crops in an agricultural catchment in South Korea
Source: PLoS One. 2018 Jan 25;13(1):e0190476. doi: 10.1371/journal.pone.0190476 (PMC5784906; doi:10.1371/journal.pone.0190476)
Supplement: S4 Table — (PDF) [file pone.0190476.s008.pdf]

	S1	S2	S3	S4
accuracy	0.93	0.93	0.93	0.92
G-mean	0.47	0.52	0.63	0.67
precision	0.67	0.65	0.63	0.59
recall	0.54	0.58	0.65	0.68
F-score	0.60	0.61	0.64	0.64
