# Supplementary material for: Classification of rare land cover types: Distinguishing annual and perennial crops in an agricultural catchment in South Korea
Source: PLoS One. 2018 Jan 25;13(1):e0190476. doi: 10.1371/journal.pone.0190476 (PMC5784906; doi:10.1371/journal.pone.0190476)
Supplement: S5 Table — (PDF) [file pone.0190476.s009.pdf]

F-score

Scenario	Min	Q_{25}	Q_{50}	Mean	Q_{75}	Max
S1	0.58	0.58	0.59	0.59	0.60	0.61
S2	0.59	0.59	0.60	0.60	0.61	0.62
S3	0.59	0.60	0.62	0.62	0.63	0.65
S4	0.62	0.62	0.63	0.63	0.64	0.64

G-mean

Scenario	Min	Q_{25}	Q_{50}	Mean	Q_{75}	Max
S1	0.44	0.44	0.45	0.45	0.46	0.48
S2	0.47	0.48	0.49	0.49	0.51	0.53
S3	0.56	0.57	0.60	0.60	0.61	0.63
S4	0.63	0.64	0.66	0.66	0.67	0.68

Precision

Scenario	Min	Q_{25}	Q_{50}	Mean	Q_{75}	Max
S1	0.64	0.65	0.66	0.66	0.66	0.69
S2	0.62	0.64	0.65	0.64	0.65	0.66
S3	0.59	0.60	0.61	0.61	0.63	0.64
S4	0.58	0.58	0.59	0.59	0.60	0.60

Recall

Scenario	Min	Q_{25}	Q_{50}	Mean	Q_{75}	Max
S1	0.52	0.53	0.53	0.53	0.54	0.54
S2	0.54	0.55	0.56	0.56	0.57	0.58
S3	0.59	0.61	0.63	0.62	0.63	0.65
S4	0.65	0.66	0.67	0.67	0.69	0.70
