# Supplementary material for: Classification of rare land cover types: Distinguishing annual and perennial crops in an agricultural catchment in South Korea
Source: PLoS One. 2018 Jan 25;13(1):e0190476. doi: 10.1371/journal.pone.0190476 (PMC5784906; doi:10.1371/journal.pone.0190476)
Supplement: S6 Table — (PDF) [file pone.0190476.s010.pdf]

| | *TPR* | | | | *FPR* | | | |
|---|---|---|---|---|---|---|---|---|
| | S1 | S2 | S3 | S4 | S1 | S2 | S3 | S4 |
| deciduous forest | 93.50 | 91.25 | 89.70 | 81.75 | 16.15 | 11.00 | 9.40 | 3.45 |
| dry field | 68.00 | 72.30 | 64.00 | 64.60 | 10.85 | 12.25 | 9.70 | 11.30 |
| paddy rice | 79.65 | 80.70 | 80.35 | 80.35 | 2.75 | 2.75 | 2.75 | 2.80 |
| fallow | 28.00 | 31.70 | 41.50 | 52.45 | 2.05 | 2.80 | 4.75 | 7.65 |
| perennial crops | 27.15 | 27.15 | 45.70 | 45.70 | 0.70 | 0.75 | 1.50 | 1.55 |
| mixed forest | 22.70 | 34.10 | 54.50 | 79.55 | 0.30 | 0.60 | 1.30 | 2.95 |
