# Supplementary material for: Classification of rare land cover types: Distinguishing annual and perennial crops in an agricultural catchment in South Korea
Source: PLoS One. 2018 Jan 25;13(1):e0190476. doi: 10.1371/journal.pone.0190476 (PMC5784906; doi:10.1371/journal.pone.0190476)
Supplement: S1 Appendix — (PDF) [file pone.0190476.s011.pdf]

List of R packages

Rasterization of the LULC data: geometry engine `GEOS` (GEOS Development Team, 2014) and the package `rgeos` (Bivand and Rundel, 2014).

SMOTE: own R functions

The removal of Tomek links: `unbalanced` (Pozzolo, Caelen, and Bontempi, 2014).

Calculation of mutual information: `parmigene` (Sales and Romualdi, 2012).

Random Forest: `randomForest` version 4.6–7 (Liaw and Wiener, 2002).

Calculation of path density: `SDMTools` (VanDerWal et al., 2014).

References

Bivand, Roger and Colin Rundel (2014). *rgeos: Interface to Geometry Engine - Open Source (GEOS)*. R package version 0.3-4.

GEOS Development Team (2014). *GEOS - Geometry Engine, Open Source*. Open Source Geospatial Foundation.

Liaw, Andy and Matthew Wiener (2002). "Classification and Regression by randomForest". In: *R news* 2.3, pp. 18–22.

Pozzolo, Andrea Dal, Olivier Caelen, and Gianluca Bontempi (2014). *unbalanced: Racing for Unbalanced Methods Selection*. R package version 1.1.

Sales, Gabriele and Chiara Romualdi (2012). *parmigene: Parallel Mutual Information estimation for Gene Network reconstruction.* R package version 1.0.2. URL: https://CRAN.R-project.org/package=parmigene.

VanDerWal, Jeremy et al. (2014). *SDMTools: Species Distribution Modelling Tools: Tools for processing data associated with species distribution modelling exercises*. R package version 1.1-221. URL: https://CRAN.R-project.org/package=SDMTools.
